# Two randomized, double-blind, placebo-controlled, dose-escalation phase 1 studies evaluating BTH1677, a 1, 3–1,6 beta glucan pathogen associated molecular pattern, in healthy volunteer subjects

**DOI:** 10.1007/s10637-016-0325-z

**Published:** 2016-02-11

**Authors:** C. E. Halstenson, T. Shamp, M. A. Gargano, R. M. Walsh, M. L. Patchen

**Affiliations:** Prism Research, 1000 Westgate Drive, St. Paul, MN 55114 USA; Biothera Pharmaceutical Inc., 3388 Mike Collins Drive, Eagan, MN 55121 USA; Respiratory Consultants, 3366 Oakdale Avenue N, Robbinsdale, MN 55422 USA

**Keywords:** BTH1677, Imprime PGG, Beta glucan, Pathogen associated molecular pattern, Pharmacokinetics, Safety

## Abstract

*Background* BTH1677 is a beta glucan pathogen associated molecular pattern (PAMP) currently being investigated as a novel cancer therapy. Here, the initial safety and pharmacokinetic (PK) results of BTH1677 in healthy subjects are reported. *Subjects and Methods* In the Phase 1a single-dosing study, subjects were randomized (3:1 per cohort) to a single intravenous (iv) infusion of BTH1677 at 0.5, 1, 2, 4, or 6 mg/kg or placebo, respectively. In the Phase 1b multi-dosing study, subjects were randomized (3:1 per cohort) to 7 daily iv infusions of BTH1677 at 1, 2, or 4 mg/kg or placebo, respectively. Safety and PK non-compartmental analyses were performed. *Results* Thirty-six subjects (*N* = 24 Phase 1a; *N* = 12 Phase 1b) were randomized to treatment. No deaths or serious adverse events occurred in either study. Mild or moderate adverse events (AEs) occurred in 67 % of BTH1677-treated subjects in both studies. Treatment-related AEs (occurring in ≥10 % of subjects) included dyspnea, flushing, headache, nausea, paraesthesia, and rash in Phase 1a and conjunctivitis and headache in Phase 1b. BTH1677 serum concentration was linear with dose. Clearance, serum elimination half-life (t_1/2_) and volume of distribution (Vss) were BTH1677 dose-independent. In Phase 1b, area under the curve, t_1/2_, and Vss values were larger at steady state on days 6–30 versus day 0. *Conclusions* BTH1677 was well tolerated after single doses up to 6 mg/kg and after 7 daily doses up to 4 mg/kg.

## Introduction

Microbial beta glucans are polymers of glucose extractable from yeasts and fungi [1]. These conserved molecules function as pathogen associated molecular patterns (PAMPs) that are efficiently recognized by the mammalian innate immune system as non-self molecules and assist in elimination of microbial pathogens [2, 3]. PAMP recognition drives enhanced killing capacity of innate immune effector cells (e.g., macrophages, monocytes, neutrophils), repolarization of macrophages from an M2 to a more M1 state, and the maturation and activation of dendritic cells—professional antigen-presenting cells that cross talk to cells of the adaptive immune system (i.e., T-cells, B-cells). BTH1677 (Biothera Pharmaceutical Inc., Eagan, MN, USA) is an intravenous (iv) formulation of a yeast-derived, uncharged, water-soluble, 1,3–1,6 beta glucan purified from the cell wall of a proprietary, non-recombinant, strain of *Saccharomyces cerevisiae*. BTH1677 is a PAMP that can orchestrate a coordinated anti-cancer immune response, involving both the innate and adaptive immune system, when administered with other anti-cancer therapies.

When BTH1677 enters the blood, it is bound by endogenous plasma anti-beta glucan antibodies (ABA) resulting in complement activation and opsonization with complement protein iC3b [4]. The BTH1677/ABA/iC3b complex initially binds to innate immune effector cells through complement receptor 3 and Fc gamma receptor IIA (FcγIIA) [4–6], activating innate immune cell function and direct killing of antibody-targeted tumor cells [7, 8]. BTH1677 also enables re-education of the tumor microenvironment, shifting the normally suppressive M2-state macrophages to a more M1 (tumor attack) state, [9, 10] and promotes depletion and/or maturation of myeloid-derived suppressor cells in the tumor microenvironment [11]. BTH1677 treatment additionally activates antigen-presenting cells, driving co-stimulatory marker expression on macrophages and dendritic cells, as well as dendritic cell maturation, CD4 and CD8 T-cell expansion, and production of key anti-tumor cytokines (e.g., interferon gamma) [10, 12–15]. In murine syngeneic and xenogeneic tumor models, the administration of BTH1677 with various tumor-targeting monoclonal antibodies has resulted in greater suppression of tumor growth and longer survival than with either agent alone [16–18]. Similar effects have been observed with BTH1677 combined with anti-angiogenic [11, 19, 20] and checkpoint inhibitor [13] antibodies.

Here, we report the initial safety and pharmacokinetic (PK) results of dose-escalating single- and multiple-dose Phase 1 studies of BTH1677 in healthy volunteer subjects.

## Materials and methods

### Study objectives

The primary objective of each study was to evaluate the safety profile and tolerability of BTH1677 versus placebo by assessing the frequency, type, and severity of adverse events (AEs), as well as by assessing changes in physical exam findings, vital signs, electrocardiogram (ECG) responses, and clinical laboratory results. The secondary objective of each study was to evaluate the PK profile of BTH1677. The Phase 1b study was initiated following completion of the Phase 1a study.

### Subjects

Subjects, 18 to 45 years of age (inclusive), were eligible for study inclusion if they provided written informed consent and had a body weight of 45 to 125 kg (inclusive), and a body mass index ≤30 kg/m^2^. Females could not be pregnant or nursing and, if premenopausal, were required to have a negative urine pregnancy test prior to enrollment and be practicing at least 2 methods of birth control. All subjects were determined to be healthy by the principal investigator on the basis of medical history, physical examination, ECG, and clinical laboratory test results. Subjects enrolled in the study understood the study requirements, agreed to abide by the study restrictions and to return for required assessments, and provided written authorization for use and disclosure of protected health information.

Subjects were excluded from the study if they had either a known hypersensitivity to baker’s yeast; a history of tobacco use within 3 months of the screening period; an active yeast infection, a positive hepatitis B, hepatitis C, or human immunodeficiency virus test (conducted during the screening period); or were a known or suspected abuser of alcohol or other drugs/substances of abuse. In addition, subjects were excluded if they had donated or lost more than a unit of blood within 30 days of the last day of the screening period; had any clinical condition that, in the opinion of the principal investigator, warranted exclusion from the study for either a scientific, procedural, or safety perspective; had taken any prescription medication within 14 days (30 days for Phase 1b); had taken any over-the-counter medication, herbal preparation, or vitamin within 7 days (acetaminophen [maximum 3 g/day], female hormone replacement therapy, and oral contraceptives were allowed); or had participated in an investigational drug study within 30 days or 5 half-lives (whichever was longer). Phase 1b also excluded subjects who had ever participated in a study with BTH1677.

### Study design

Both Phase 1a (NCT00542217) and Phase 1b (NCT00542464) were single-center, randomized, double-blind, placebo-controlled, dose-escalation studies that evaluated infusion of BTH1677 in healthy volunteer subjects.

The Phase 1a, single-dosing study consisted of a 1-day treatment period and an additional 6-day follow-up period that enrolled 24 subjects in 5 sequential BTH1677 dose cohorts (0.5, 1, 2, 4, and 6 mg/kg). The Phase 1b multiple-dosing study consisted of a 7-day treatment period and an additional 23-day follow-up period and enrolled 12 subjects in 3 sequential dose cohorts (1, 2, and 4 mg/kg). The initial BTH1677 dose chosen for the Phase 1a study was based on safety margins calculated from animal toxicology studies according to regulatory guidelines [21]. For each cohort, in a double-blind manner, 3 subjects were randomized to BTH1677 and 1 subject was randomized to placebo. AEs were assessed as mild—awareness of sign, symptom, or event, but easily tolerated; moderate—discomfort enough to cause interference with usual activities and may warrant intervention; severe—incapacitating with inability to do usual activities or significantly affecting clinical status, and warranting intervention; or life threatening—immediate risk of death. Each dose increase was dependent on the approval of an AE taskforce (AETF), based on review of safety data from the previous cohort. The AETF consisted of the principal investigator and 3 members of the Biothera clinical team, including the medical monitor. Dose escalation of study drug was to be discontinued if dose-limiting toxicity (DLT) was observed in the previous cohort. DLT was defined as 2 or more subjects experiencing at least 1 moderate AE judged to be study drug related or 1 or more subjects experiencing at least 1 severe AE or serious AE (SAE) judged to be study drug related.

**Fig. 1:**
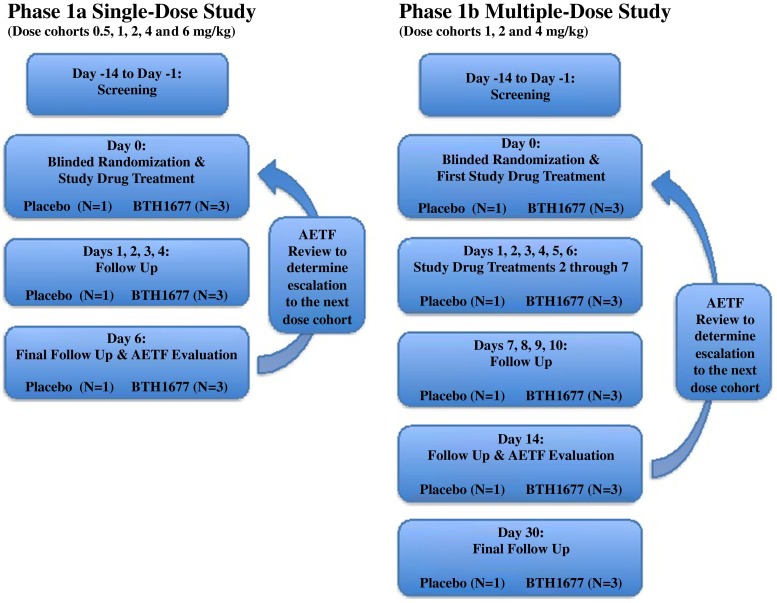
Phase 1a single-dose (left) and Phase 1b multiple-dose (right) BTH1677 study designs. Abbreviation: AETF, Adverse event taskforce

It was the original intent of the Phase 1a protocol to dose all subjects over a 1-h (hr) period; however, during the study the protocol was amended to revise dosing times resulting in enrollment of an additional 4 subjects to an amended dosing schedule in the 4 mg/kg cohort (see additional information provided in the Results section). Planned dose cohorts and infusion information for the Phase 1a and Phase 1b studies are shown in Table 1. Figure 1 illustrates the design of the Phase 1a and Phase 1b studies.

**Table 1:** Target drug infusion plans for the Phase 1a single-dose and Phase 1b multiple-dose BTH1677 clinical studies

BTH1677 Dose Level (mg/kg)	Dosed in Phase 1a	Dosed in Phase 1b	Total Infusion Volume (mL)	BTH1677 Concentration in Infusion Volume (mg/mL)^a^	Infusion Time (hr)	Rate of Infusion (mg/min)^a^
0.5	Yes	No	250	0.15	1	0.63
1.0	Yes	Yes	250	0.3	1	1.25
2.0	Yes	Yes	250	0.6	1	2.50
4.0	Yes	No	500	0.6	1	5.00
	Yes	Yes	500	0.6	2	2.50
6.0	Yes	No	750	0.6	3	2.50

^a^Concentration and rate calculations are based on the average subject weight of 75 kg and are approximate; actual values based on total body weight differed among subjects.

### BTH1677

Biothera Pharmaceutical Inc. (Eagan, MN, USA) supplied BTH1677 (Imprime PGG; β(1,6)-[poly-(1,3)-D-glucopyranosyl]-poly-β-(1,3)-D-glucopyranose) to the clinical site in 20-mL glass vials at a concentration of 1 mg/mL in 0.14 M sodium chloride and 0.11 M sodium citrate. The average molecular weight of BTH1677 is approximately 150,000 Da and the empirical formula is (C_6_H_10_O_5_)_n_. For infusion, BTH1677 was diluted at the clinical site in 0.9 % normal saline.

### Pharmacokinetic analysis

In both studies, serum BTH1677 levels were analyzed using an enzyme-linked immunosorbent assay (ELISA) method that was qualified at Biothera Pharmaceutical Inc. (Eagan, MN, USA). The assay was based on galactosylceramide (GalCer; CAS #85305–88-0) capture of BTH1677 and detection with a beta-glucan-specific IgM monoclonal antibody (BFD IV). Test serum samples were incubated in 96-well microtiter plates coated with GalCer to allow for BTH1677 binding to the GalCer coating. The bound BTH1677 was then detected by sequential incubations with unlabeled BFD IV, then biotin-conjugated goat anti-mouse IgM, and finally with streptavidin-conjugated horseradish peroxidase and peroxidase substrate. Color development was quenched by adding the substrate stop solution to each well and absorbance was read at 450 nm. The lower limit of detection for the assay was 1.2 ng/mL and the lower limit of quantitation was 4.7 ng/mL. For PK analysis in the Phase 1a study, blood samples were collected before dosing and at 1, 1.25, 1.5, 2, 3, 4, 8, 12, 24, 48, 72, 96, and 168 h relative to the start of the infusion. For PK analysis in the Phase 1b study, on the first day (day 0) and last day (day 6) of dosing, blood samples were collected before dosing, at the end of infusion, and at 0.25, 0.5, 1, 2, 3, 7, and 12 h relative to the end of infusion. Additional blood samples were collected at 24, 48, 72, 96, 120, 168, 192, 216, 240, 336, and 720 h relative to the start of infusion on day 0. After blood collection, serum was separated, frozen, and analyzed for BTH1677 concentrations. For both studies, PK parameters were calculated using non-compartmental analysis (NCA) methods.

### Statistical analysis

Because of the nature of the Phase 1a and Phase 1b studies, statistical analyses were descriptive only. In each study, data for all subjects randomized to placebo were pooled to create the placebo treatment group. Categorical data were summarized in tables listing the frequency and the percentage of subjects in each treatment group. Continuous data were summarized in tables listing the mean, standard deviation, median, minimum, and maximum in each treatment group. All statistical computations were performed using the SAS® system.

## Results

### Phase 1a study

#### Subject demographics

Baseline demographics of subjects in the Phase 1a single-dosing study are shown on the left-hand side of Table 2. Overall, the majority of the population in both treatment groups was male (72.2 % BTH1677; 100 % placebo) and Caucasian (72.2 % BTH1677; 83.3 % placebo). The mean age was 25.1 years (range, 18–44 years) for subjects receiving BTH1677 and 28.0 years (range, 21–43 years) for subjects receiving placebo.

**Table 2:** Subject demographics by study and treatment group

	Phase 1a Single-Dose Study Treatment Groups									Phase 1b Multiple-Dose Study Treatment Groups				
	BTH1677								Placebo	BTH1677				Placebo
	0.5 mg/kg (1-h infusion) (*n* = 3)	1 mg/kg (1-h infusion) (*n* = 3)	2 mg/kg (1-h infusion) (*n* = 4)	4 mg/kg (1-h infusion) (*n* = 2)	4 mg/kg (2-h infusion) (*n* = 3)	6 mg/kg (3-h infusion) (*n* = 3)	All BTH1677 (*n* = 18)	All BTH1677 Minus 4 mg/kg 1-h Infusion Group^a^ (*n* = 16)	All Placebo^b^ (*n* = 6)	1 mg/kg (1-h infusion) (*n* = 3)	2 mg/kg (1-h infusion) (*n* = 3)	4 mg/kg (2-h infusion) (*n* = 3)	All BTH1677 (*n* = 9)	All Placebo^b^ (*n* = 3)
Sex^c^														
Male	2 (66.7)	3 (100.0)	3 (75.0)	2 (100.0)	2 (66.7)	1 (33.3)	13 (72.2)	11 (68.8)	6 (100.0)	1 (33.3)	1 (33.3)	2 (66.7)	4 (44.4)	1 (33.3)
Female	1 (33.3)	0 (0.0)	1 (25.0)	0 (0.0)	1 (33.3)	2 (66.7)	5 (27.8)	5 (31.2)	0 (0.0)	2 (66.7)	2 (66.7)	1 (33.3)	5 (55.6)	2 (66.7)
Race^c^														
Caucasian	3 (100.0)	1 (33.3)	3 (75.0)	2 (100.0)	2 (67.7)	2 (67.7)	13 (72.2)	11 (68.8)	5 (83.3)	2 (66.7)	3 (100.0)	3 (100.0)	8 (88.9)	2 (66.7)
African-American	0 (0.0)	1 (33.3)	0 (0.0)	0 (0.0)	1 (33.3)	1 (33.3)	3 (16.7)	3 (18.8)	1 (16.7)	0 (0.0)	0 (0.0)	0 (0.0)	0 (0.0)	0 (0.0)
Hispanic	0 (0.0)	1 (33.3)	0 (0.0)	0 (0.0)	0 (0.0)	0 (0.0)	1 (5.6)	1 (6.2)	0 (0.0)	1 (33.3)	0 (0.0)	0 (0.0)	1 (11.1)	1 (33.3)
Asian	0 (0.0)	0 (0.0)	1 (25.0)	0 (0.0)	0 (0.0)	0 (0.0)	1 (5.6)	1 (6.2)	0 (0.0)	0 (0.0)	0 (0.0)	0 (0.0)	0 (0.0)	0 (0.0)
Age (years)														
Mean	27.7	27.0	27.5	19.5	26.3	23.0	25.1	26.4	28.0	26.0	21.7	36.3	28.0	23.7
SD^d^	12.5	8.7	11.2	2.1	10.4	3.6	8.6	8.7	8.8	12.1	2.1	10.8	10.5	4.0
Median	22.0	23.0	23.5	19.5	23.0	22.0	22.4	23.0	24.5	19.0	21.0	41.0	24.0	23.0
Min, Max	19, 42	21, 37	19, 44	18, 21	18, 38	20, 27	18, 44	18, 44	21, 43	19, 40	20, 24	24, 44	19, 44	20, 28
Weight (kg)														
Mean	92.37	79.43	79.70	91.30	67.33	72.23	79.75	78.31	84.48	67.50	65.70	81.27	71.49	67.30
SD^d^	12.208	23.133	20.112	8.202	10.748	21.134	16.585	17.856	23.531	6.062	8.072	9.139	10.037	16.543
Median	86.50	83.60	80.50	91.30	72.30	66.30	79.48	79.15	80.45	64.00	68.10	83.90	71.10	58.00
Min	84.2	54.5	56.6	85.5	55.0	54.7	55.5	54.5	61.7	64.0	56.7	71.1	56.7	57.5
Max	106.4	100.2	101.2	97.1	74.7	95.7	106.4	106.4	122.7	74.5	72.3	88.8	88.8	86.4

*Min* minimum, *Max* maximum

^a^During the Phase 1a study, infusion rate was shown to have an effect on infusion-related AEs which became evident at the 4 mg/kg dose. This resulted in extending infusion time from 1 h to 2 h for a second 4-mg/kg cohort and to 3 h for the 6-mg/kg cohort. This subgroup analysis eliminates the 4-mg/kg 1-h infusion subjects shown to have AEs due to the infusion rate.

^b^For subjects receiving placebo, infusion time matched that of the corresponding treatment group.

^c^Data presented as the number (percent) of subjects.

^d^Standard deviation.

#### BTH1677 drug administration

In this study, 24 subjects were randomized and dosed with single doses of placebo (*n* = 6) or escalating doses of BTH1677 at 0.5, 1, 2, 4, or 6 mg/kg (*n* = 18). It was originally intended in the protocol to dose all subjects over a 1-h period. However, after initiating dosing of 3 subjects in the 4-mg/kg cohort, 2 subjects reported moderate AEs of flushing, pruritus, tachycardia, dyspnea, and hyperhidrosis within 5 to 10 min of initiation of infusion. At the onset of the AEs, study drug administration was stopped in all 3 subjects (which included 1 subject randomized to placebo who had experienced no AEs) and initiation of dosing in the fourth subject planned for this cohort (a subject randomized to BTH1677) was suspended. The dosed subjects experiencing AEs were administered diphenhydramine (i.e., Benadryl®) intravenously and their AEs resolved within approximately 10 to 15 min after discontinuation of the infusion. At the time study drug administration was discontinued in the 4-mg/kg (1-h infusion) treatment cohort, the subjects exhibiting the abovementioned AEs had received approximately 9 % to 14 % of the total targeted infusion volume. Thus, the total study drug dose administered to these subjects was far less than the total dose safely administered to the 2-mg/kg treatment cohort. Although the 4-mg/kg (1-h infusion) dose was diluted in a 500-mL volume of normal saline versus the 250-mL volume of normal saline used for the previous 2-mg/kg dose, the infusion time was kept constant at 1 h. As a result, the concentration of the study drug infused was similar between the 4-mg/kg (1-h infusion) and 2-mg/kg treatment cohorts (mean concentration of 0.64 mg/mL for the 2-mg/kg treatment cohort compared to 0.73 mg/mL for the 4-mg/kg [1-h infusion] treatment cohort). However, the rate of administration of the 4-mg/kg (1-h infusion) dose was approximately twice that of 2-mg/kg dose. The rate of drug administration for the 4-mg/kg (1-h infusion) cohort varied from 5.7 to 6.5 mg/min, whereas the rate of drug administration for the 2-mg/kg cohort varied from 1.9 to 3.4 mg/min.

After review of the clinical and safety data, it was concluded that the AEs observed at the 4-mg/kg (1-h infusion) dose level were most likely not the result of the BTH1677 drug dose, but rather were a result of the rapid infusion rate and were not considered DLTs. A protocol amendment was submitted and approved to allow enrollment of 4 additional subjects into the 4-mg/kg treatment cohort with a 2-h, rather than 1-h, infusion time to allow an infusion rate similar to that of the previous 2-mg/kg treatment cohort in which BTH1677 had been well tolerated. In support of the conclusion that the observed AEs were the result of infusion rate rather than dose level, there were no treatment-related AEs reported in subjects treated with 4 mg/kg BTH1677 administered over a 2-h infusion. The protocol amendment also modified the infusion time for the 6-mg/kg dose cohort to 3 h, which was also generally well tolerated.

#### Safety

An overview of the safety summary is shown on the left side of Table 3. Twelve of the 18 BTH1677 subjects (66.7 %) and 3 of 6 placebo subjects (50.0 %) experienced AEs. AEs were assessed as treatment related in 9 of 18 BTH1677 subjects (50.0 %) and in none of the 6 placebo subjects (0.0 %). All AEs were mild or moderate in intensity, with no AEs classified as severe. No SAEs or deaths occurred in the study. Excluding the 4-mg/kg 1-h infusion group, the mean number of treatment-related AEs per BTH1677 subject in the 0.5-, 1-, 2- and 4-mg/kg groups ranged from 0.0 to 1.0 and increased to 2.7 in the 6-mg/kg group. Four subjects (2 in the 4-mg/kg 1-h infusion group) discontinued the study prematurely because of AEs that were considered treatment related; these AEs are further discussed below.

**Table 3:** Overall safety summary by study and treatment group

	Phase 1a Single-Dose Study Treatment Groups									Phase 1b Multiple-Dose Study Treatment Groups				
	BTH1677								Placebo	BTH1677				Placebo
	0.5 mg/kg (1-h infusion) (*n* = 3)	1 mg/kg (1-h infusion) (*n* = 3)	2 mg/kg (1-h infusion) (*n* = 4)	4 mg/kg (1-h infusion) (*n* = 2)	4 mg/kg (2-h infusion) (*n* = 3)	6 mg/kg (3-h infusion) (*n* = 3)	All BTH1677 (*n* = 18)	All BTH1677 Minus 4 mg/kg 1-h Infusion Group^a^ (*n* = 16)	All Placebo^b^ (*n* = 6)	1 mg/kg (1-h infusion) (*n* = 3)	2 mg/kg (1-h infusion) (*n* = 3)	4 mg/kg (2-h infusion) (*n* = 3)	All BTH1677 (*n* = 9)	All Placebo^b^ (*n* = 3)
Subjects with AEs, n (%)^c^	3 (100.0)	1 (33.3)	2 (50.0)	2 (100.0)	1 (33.3)	3 (100.0)	12 (66.7)	10 (62.5)	3 (50.0)	1 (33.3)	2 (66.7)	3 (100.0)	6 (66.7)	2 (66.7)
Mild	3 (100.0)	1 (33.3)	0 (0.0)	0 (0.0)	1 (33.3)	2 (66.7)	7 (38.9)	7 (43.8)	1 (16.7)	1 (33.3)	0 (0.0)	1 (33.3)	2 (22.2)	0 (0.0)
Moderate	0 (0.0)	0 (0.0)	2 (50.0)	2 (100.0)	0 (0.0)	1 (33.3)	5 (27.8)	3 (18.8)	2 (33.3)	0 (0.0)	2 (66.7)	2 (66.7)	4 (44.4)	2 (66.7)
Severe	0 (0.0)	0 (0.0)	0 (0.0)	0 (0.0)	0 (0.0)	0 (0.0)	0 (0.0)	0 (0.0)	0 (0.0)	0 (0.0)	0 (0.0)	0 (0.0)	0 (0.0)	0 (0.0)
AEs, n	4	1	3	10	2	8	28	18	10^e^	3	2	3	8	3
Mean AEs per subject	1.3	1.0	1.5	5.0	2.0	2.7	2.3	1.1	3.3^e^	3.0	1.0	1.0	1.3	1.5
SAEs, n (%)	0 (0.0)	0 (0.0)	0 (0.0)	0 (0.0)	0 (0.0)	0 (0.0)	0 (0.0)	0 (0.0)	0 (0.0)	0 (0.0)	0 (0.0)	0 (0.0)	0 (0.0)	0 (0.0)
Deaths, n (%)	0 (0.0)	0 (0.0)	0 (0.0)	0 (0.0)	0 (0.0)	0 (0.0)	0 (0.0)	0 (0.0)	0 (0.0)	0 (0.0)	0 (0.0)	0 (0.0)	0 (0.0)	0 (0.0)
Subjects who discontinued due to AEs, n (%)	0 (0.0)	0 (0.0)	1 (25.0)	2 (100.0)	0 (0.0)	1 (33.3)	4 (22.2)	2 (12.5)	0 (0.0)	0 (0.0)	0 (0.0)	1 (33.3)	1 (11.1)	1 (33.3)
Subjects with AEs assessed as treatment related, n (%)^c^^,^^d^	2 (66.7)	1 (33.3)	1 (25.0)	2 (100.0)	0 (0.0)	3 (100.0)	9 (50.0)	7 (43.8)	0 (0.0)	0 (0.0)	0 (0.0)	2 (66.7)	2 (22.2)	1 (33.3)
Mild	2 (66.7)	1 (33.3)	0 (0.0)	0 (0.0)	0 (0.0)	2 (66.7)	5 (27.8)	5 (31.3)	0 (0.0)	0 (0.0)	0 (0.0)	1 (33.3)	1 (11.1)	0 (0.0)
Moderate	0 (0.0)	0 (0.0)	1 (25.0)	2 (100.0)	0 (0.0)	1 (33.3)	4 (22.2)	2 (12.5)	0 (0.0)	0 (0.0)	0 (0.0)	1 (33.3)	1 (11.1)	1 (33.3)
Severe	0 (0.0)	0 (0.0)	0 (0.0)	0 (0.0)	0 (0.0)	0 (0.0)	0 (0.0)	0 (0.0)	0 (0.0)	0 (0.0)	0 (0.0)	0 (0.0)	0 (0.0)	0 (0.0)
AEs assessed as treatment related, n	2	1	1	9	0	8	21	12	0	0	0	2	2	1
Mean treatment-related AEs per subject	1.0	1.0	1.0	4.5	0.0	2.7	2.3	1.7	0.0	0.0	0.0	1.0	1.0	1.0

*AE* adverse events

^a^During the Phase 1a study, infusion rate was shown to have an effect on infusion-related AEs which became evident at the 4-mg/kg dose. This resulted in extending infusion time from 1 h to 2 h for a second 4-mg/kg cohort and to 3 h for the 6-mg/kg cohort. This subgroup analysis eliminates the 4-mg/kg 1-h infusion subjects shown to have AEs due to the infusion rate.

^b^For subjects receiving placebo, infusion time matched that of the corresponding treatment group.

^c^A single subject may have multiple AEs with different intensities; only the most severe intensity is included in this count.

^d^Relatedness definitions differed between the studies; Phase 1a used not related, related, and unknown and Phase 1b used unrelated, unlikely related, possibly related, probably related, definitely related, and unknown. Treatment-related AEs include AEs assessed by the investigator as related for Phase 1a and possibly, probably, or definitely related for Phase1b.

^e^A single subject in this group reported 8 adverse events.

The highest frequency of AEs was reported among subjects receiving 6-mg/kg BTH1677 over a 3-h infusion period (Table 4). AEs reported by more than 1 subject included headache (4 events; 2 events in BTH1677 subjects [both mild] and 2 events in placebo subjects [1 mild and 1 moderate]), dyspnea (3 events in BTH1677 subjects [1 mild and 2 moderate]), and 2 events each of nausea [1 mild and 1 moderate], paraesthesia [both mild], rash [both moderate] and flushing [both moderate] in BTH1677 subjects.

**Table 4:** Phase 1a single-dose BTH1677 study: Subjects with all and treatment-related adverse events by treatment group

System Organ Class and Preferred Term ^a,b^	Treatment Group							
	BTH1677							Placebo
	0.5 mg/kg (1-h infusion) (*n* = 3)	1 mg/kg (1-h infusion) (*n* = 3)	2 mg/kg (1-h infusion) (*n* = 4)	4 mg/kg (1-h infusion) (*n* = 2)	4 mg/kg (2-h infusion) (*n* = 3)	6 mg/kg (3-h infusion) (*n* = 3)	All BTH1677 (*n* = 18)	All Placebo^c^ (*n* = 6)
**Subjects with any AE/Treatment-related AE**	**3/2**	**1/1**	**2/1**	**2/2**	**1/0**	**3/3**	**12/9**	**3/0**
**Cardiac disorders**	**0/0**	**0/0**	**0/0**	**1/1**	**0/0**	**0/0**	**1/1**	**0/0**
Tachycardia	0/0	0/0	0/0	1/1^d^	0/0	0/0	1/0	0/0
**Eye disorders**	**0/0**	**0/0**	**0/0**	**0/0**	**0/0**	**0/0**	**0/0**	**1/0**
Photophobia	0/0	0/0	0/0	0/0	0/0	0/0	0/0	1/0
**Gastrointestinal disorders**	**0/0**	**0/0**	**0/0**	**1/1**	**0/0**	**1/1**	**2/2**	**1/0**
Nausea	0/0	0/0	0/0	1/1^d^	0/0	1/1	2/2	0/0
Diarrhea	0/0	0/0	0/0	0/0	0/0	0/0	0/0	1/0
Flatulence	0/0	0/0	0/0	0/0	0/0	0/0	0/0	1/0
Gingival pain	0/0	0/0	0/0	0/0	0/0	0/0	0/0	1/0
Toothache	0/0	0/0	0/0	0/0	0/0	0/0	0/0	1/0
**General disorders and administration site conditions**	**0/0**	**1/1**	**0/0**	**0/0**	**0/0**	**2/2**	**3/3**	**0/0**
Chest discomfort	0/0	0/0	0/0	0/0	0/0	1/1	1/1	0/0
Chills	0/0	0/0	0/0	0/0	0/0	1/1	1/1	0/0
Infusion site pain	0/0	1/1	0/0	0/0	0/0	0/0	1/1	0/0
**Infections and infestations**	**1/0**	**0/0**	**0/0**	**0/0**	**0/0**	**0/0**	**1/0**	**0/0**
Upper respiratory tract infection	1/0	0/0	0/0	0/0	0/0	0/0	1/0	0/0
**Injury, poisoning and procedural complications**	**0/0**	**0/0**	**1/0**	**0/0**	**1/0**	**0/0**	**2/0**	**0/0**
Back injury	0/0	0/0	1/0	0/0	0/0	0/0	1/0	0/0
Laceration	0/0	0/0	0/0	0/0	1/0	0/0	1/0	0/0
**Investigations**	**0/0**	**0/0**	**0 /0**	**1/0**	**0/0**	**0/0**	**1/0**	**0/0**
White blood cell count decreased	0/0	0/0	0/0	1/0	0/0	0/0	1/0	0/0
**Musculoskeletal and connective tissue disorders**	**0/0**	**0/0**	**1/0**	**0/0**	**0/0**	**0/0**	**1/0**	**0/0**
Arthralgia	0/0	0/0	1/0	0/0	0/0	0/0	1/0	0/0
**Nervous system disorders**	**2/2**	**0/0**	**0/0**	**1/1**	**0/0**	**2/2**	**5/5**	**2/0**
Headache	2/2	0/0	0/0	0/0	0/0	0/0	2/2	2/0
Dizziness	0/0	0/0	0/0	1/1^d^	0/0	0/0	1/1	1/0
Paraesthesia	0/0	0/0	0/0	0/0	0/0	2/2^d^	2/2	0/0
Hypoaesthesia	0/0	0/0	0/0	0/0	0/0	1/1^d^	1/1	0/0
Migraine	0/0	0/0	0/0	0/0	0/0	0/0	0/0	1/0
**Respiratory, thoracic and mediastinal disorders**	**1/0**	**0/0**	**0/0**	**2/2**	**0/0**	**1/1**	**4/3**	**0/0**
Dyspnea	0/0	0/0	0/0	2/2^d^	0/0	1/1^d^	3/3	0/0
Throat irritation	1/0	0/0	0/0	0/0	0/0	0/0	1/0	0/0
**Skin and subcutaneous tissue disorders**	**0/0**	**0/0**	**1/1**	**2/2**	**1/0**	**1/1**	**5/4**	**0/0**
Rash	0/0	0/0	1/1^d^	0/0	0/0	1/1^d^	2/2	0/0
Ecchymosis	0/0	0/0	0/0	0/0	1/0	0/0	1/0	0/0
Hyperhidrosis	0/0	0/0	0/0	1/1^d^	0/0	0/0	1/1	0/0
Pruritus	0/0	0/0	0/0	1/1^d^	0/0	0/0	1/1	0/0
**Vascular disorders**	**0/0**	**0/0**	**0/0**	**2/2**	**0/0**	**0/0**	**2/2**	**0/0**
Flushing	0/0	0/0	0/0	2/2^d^	0/0	0/0	2/2	0/0

*AE* adverse events

^a^Coded by Medical Dictionary for Regulatory Activities (MEdDRA; V 8.1) by System Organ Class (SOC) (bolded terms) and preferred term (non-bolded terms).

^b^Data are presented as the number of subjects experiencing any AE and the number of subjects experiencing treatment-related AEs separated by “/”. For both categories, each subject was counted only once for each SOC and each preferred term; a single subject may have had multiple AEs within a specific SOC. Treatment-related AEs included AEs assessed by the investigator as related from the options of not related, related, and unknown.

^c^For subjects receiving placebo, infusion time matched that of the corresponding treatment group.

^d^AEs leading to premature discontinuation from the study (paraesthesia led to discontinuation in only 1 subject).

The treatment-related AEs reported by the 9 BTH1677 subjects are also reported in Table 4. In the 4-mg/kg 1-h infusion group, all AEs but 1 (moderate decreased white blood cell count) were considered treatment related. Across all subjects, treatment-related AEs reported by more than 1 subject included dyspnea (3 events [1 mild and 2 moderate]) and 2 events each of nausea (1 mild and 1 moderate), headache (both mild), paraesthesia (both mild), rash (both moderate), and flushing (both moderate). Treatment-related AEs leading to premature study discontinuation consisted of moderate rash in a 2-mg/kg subject; moderate tachycardia, moderate nausea, moderate dizziness, moderate dyspnea, moderate hyperhidrosis, moderate pruritus and moderate flushing in the two 4-mg/kg 1-h infusion subjects (only dyspnea and flushing were experienced by both subjects); and mild paraesthesia, mild hypoaesthesia, and moderate rash in a 6-mg/kg subject. Overall, moderate treatment-related AEs experienced by subjects other than those undergoing infusion reactions in the 4 mg/kg 1-h infusion group consisted of only 1 subject at 2 mg/kg (rash) and 1 subject at 6 mg/kg (rash), hence, DLT was not observed at any dose tested.

Overall, only minor deviations from the normal range were observed for hematology, chemistry, or urinalysis parameters at various times during the study. These deviations were observed in subjects treated with both placebo and BTH1677, and there were no consistent mean changes from baseline among the treatment groups. As indicated above, only 1 laboratory abnormality was considered clinically significant and reported as an AE. This AE, decreased white blood cell count, occurred in a subject dosed with BTH1677 in the 4-mg/kg 1-h infusion group and had resolved by the end of the study (day 6). Other than physical changes captured as AEs above, all of which had resolved by the end of the study, no additional abnormal physical examination findings were observed after BTH1677 administration nor were changes from baseline noted in vital signs or ECGs.

### Phase 1b study

#### Subject demographics

Baseline demographics of subjects in the Phase 1b multiple-dosing study are shown on the right-hand side of Table 2. The subject population was predominately female (55.6 % BTH1677; 66.7 % placebo) and included Caucasians (88.9 % BTH1677; 66.7 % placebo) and Hispanics (11.1 % BTH1677; 33.3 % placebo). The mean age was 28.0 years (range, 19–44 years) for subjects receiving BTH1677 and 23.7 years (range, 20–28 years) for subjects receiving placebo.

#### BTH1677 drug administration

Twelve subjects were randomized and dosed with 7 consecutive daily doses of BTH1677 at 1, 2, or 4 mg/kg. Based on experience from the Phase 1a study, the 1- and 2-mg/kg doses were infused over 1 h and the 4-mg/kg dose was infused over 2 h.

#### Safety

As shown on the right-hand side of Table 3, 6 of 9 BTH1677 subjects (66.7 %) and 2 of 3 placebo subjects (66.7 %) experienced AEs. AEs were assessed as treatment related in 2 of 9 BTH1677 subjects (22.2 %) and in 1 of 3 placebo subjects (33.3 %). As in the Phase 1a study, all AEs were mild or moderate in intensity. Furthermore, no SAEs or deaths occurred in the study. All AEs designated as treatment related occurred in subjects in the 4-mg/kg BTH1677 group. Two subjects discontinued the study prematurely because of AEs that were considered treatment related; these AEs are discussed further below.

The highest frequency of AEs was reported in the 4-mg/kg group (Table 5). Only 1 AE, headache, was reported by more than 1 subject; this AE was reported by 1 subject in both the BTH1677 1-mg/kg group (mild) and the BTH1677 4-mg/kg group (mild). AEs assessed as treatment-related in the Phase 1b study are also reported in Table 5. Three subjects experienced treatment-related AEs. In separate subjects in the BTH1677 4-mg/kg group, these included conjunctivitis (moderate) and headache (mild) and, in a placebo subject, moderate increase in blood creatinine kinase (CK). The conjunctivitis and increased CK led to premature study discontinuation in the subjects experiencing these events. DLT was not observed at any dose tested.

**Table 5:** Phase 1b multiple-dose BTH1677 study: Subjects with all and treatment-related adverse events by treatment group

System Organ Class and Preferred Term^a^^,^^b^	Treatment Group				
	BTH1677				Placebo
	1 mg/kg (1-h infusion) (*n* = 3)	2 mg/kg (1-h infusion) (*n* = 3)	4 mg/kg (2-h infusion) (*n* = 3)	All BTH1677 (*n* = 9)	All Placebo^c^ (*n* = 3)
**Subjects with any AE/Treatment-related AE**	**1/0**	**2/0**	**3/2**	**6/2**	**2/1**
**Blood and lymphatic system disorders**	**0/0**	**1/0**	**0/0**	**1/0**	**0/0**
Anemia	0/0	1/0	0/0	1/0	0/0
**Eye disorders**	**1/0**	**0/0**	**1/1**	**2/1**	**0/0**
Conjunctivitis	0/0	0/0	1/1^d^	1/1	0/0
Vision blurred	1/0	0/0	0/0	1/0	0/0
**General disorders and administration site conditions**	**1/0**	**0/0**	**0/0**	**1/0**	**1/0**
Fatigue	1/0	0/0	0/0	1/0	0/0
Pain	0/0	0/0	0/0	0/0	1/0
**Injury, poisoning, and procedural complications**	**0/0**	**1/0**	**0/0**	**1/0**	**0/0**
Excoriation	0/0	1/0	0/0	1/0	0/0
**Investigations**	**0/0**	**0/0**	**0/0**	**0/0**	**1/1**
Blood creatinine kinase increased	0/0	0/0	0/0	0/0	1/1^d^
**Musculoskeletal and connective tissue disorders**	**0/0**	**0/0**	**1/0**	**1/0**	**1/0**
Musculoskeletal stiffness	0/0	0/0	1/0	1/0	0/0
Myalgia	0/0	0/0	0/0	0/0	1/0
**Nervous system disorders**	**1/0**	**0/0**	**1/1**	**2/1**	**0/0**
Headache	1/0	0/0	1/1	2/1	0/0

*AE* adverse events

^a^Coded by Medical Dictionary for Regulatory Activities (MEdDRA; V 8.1) by System Organ Class (SOC) (bolded terms) and preferred term (non-bolded terms).

^b^Data are presented as the number of subjects experiencing any AE and the number of subjects experiencing treatment-related AEs separated by “/”. For both categories, each subject was counted only once for each SOC and each preferred term; a single subject may have had multiple AEs within a specific SOC. Treatment-related AEs included AEs assessed by the investigator as possibly, probably, or definitely related from the options of unrelated, unlikely related, possibly related, probably related, definitely related, and unknown.

^c^For subjects receiving placebo, infusion time matched that of the corresponding treatment group.

^d^AEs leading to premature discontinuation from the study.

No clear trends in mean changes from baseline among the treatment groups were noted for hematology, clinical chemistry, or urinalysis parameters. In addition, other than conjunctivitis captured, as an AE above and which had resolved by end of study, no additional abnormal physical examination findings or changes in vital signs from baseline were observed after BTH1677 administration. There were some abnormal ECG results (or normal ECG results with variants) observed after study drug administration compared with baseline; however, these changes were not deemed clinically significant.

### Pharmacokinetic analysis

Key PK results from the Phase 1a single-dose study based on NCA methods are shown in Table 6. BTH1677 exposure was generally linear in the range of 0.5 mg/kg to 6.0 mg/kg for single iv doses based on area under the curve (AUC) values for AUC_(0–24)_, AUC_(0-last)_, and AUC_(0-∞)_. The PK parameter for maximum drug concentration (C_max_) in this study was generally linear in the dose range of 0.5 mg/kg to 2 mg/kg, in which all subjects were dosed over 1 h, but not in the 4 mg/kg and 6 mg/kg doses given over longer periods. This was attributed to the different infusion times used among treatments. PK parameters for clearance (CL), serum elimination half-life (t_1/2_) and volume of distribution (V_ss_) were BTH1677 dose independent, but time to maximum concentration (T_max_) was dose dependent. The dose dependency of T_max_ was again attributed to the different infusion times used among treatments. T_max_ occurred at approximately 1 h for the 1-h infusion groups, 2 h for the 2-h infusion group, and 2.67 h for the 3-h infusion group. Mean CL values ranged from 441.76 mL/h to 619.25 mL/h, mean t_1/2_ values ranged from 23.36 h to 32.75 h, and mean V_ss_ values ranged from 7794.36 mL to 14,400.69 mL.

**Table 6:** Phase 1a single-dose non-compartmental pharmacokinetic parameters by treatment group

Pharmacokinetic Parameters^a^										
BTH1677 Dose (mg/kg)	Infusion Time (hr)	N	AUC_(0–24)_ (hr*mcg/mL)	AUC_(0-last)_ (hr*mcg/mL)	AUC_(0-∞)_ (hr*mcg/mL)	CL (mL/h)	C_max_ (mcg/mL)	t_1/2_ (hr)	T_max_ (hr)	V_ss_ (mL)
0.5	1.0	3	56.55 ± 9.72	74.18 ± 8.11	74.96 ± 8.12	619.25 ± 84.21	14.76 ± 4.09	28.55 ± 18.96	1.00 ± 0.00	10,330.31 ± 1717.26
1	1.0	3	119.69 ± 43.99	166.76 ± 57.12	169.53 ± 59.58	475.73 ± 31.88	29.72 ± 16.60	31.10 ± 10.00	1.08 ± 0.14	10,903.43 ± 416.17
2	1.0	3	233.04 ± 60.25	290.89 ± 76.53	292.33 ± 75.65	530.38 ± 144.79	32.83 ± 9.08	23.36 ± 8.69	1.00 ± 0.00	7794.36 ± 4131.94
4^b^	1.0	2	83.01 ± 41.98	315.73 ± 159.99	NC^c^	NC	5.94 ± 3.05	NC	1.12 ± 0.18	NC
4	2.0	3	325.43 ± 58.16	429.69 ± 65.77	442.66 ± 60.63	617.91 ± 143.58	51.24 ± 15.45	32.75 ± 12.22	2.00 ± 0.00	14,400.69 ± 7379.93
6^d^	3.0	3	459.60 ± 174.05	868.01 ± 107.06	837.59 ± 116.59	441.76 ± 120.25	53.60 ± 28.24	29.52 ± 1.55	2.67 ± 1.53	10,386.67 ± 1728.99

*AUC*_(*0–24*)_ area under the serum concentration versus time curve from time 0 to 24 h after the end of the infusion, *AUC*_(*0-last*)_ area under the serum concentration versus time curve from time 0 until last measurable concentration, *AUC*_(*0-∞*)_ area under the serum concentration versus time curve from time 0 extrapolated to infinity, *CL* clearance, *C*_*max*_ maximum concentration, *t*_*1*/*2*_ terminal elimination half-life, *T*_*max*_ time of maximum concentration, *V*_*ss*_ steady state of volume distribution

^a^Mean values ± standard deviation.

^b^Less than 75 % of planned values collected for all subjects.

^c^Not Calculated.

^d^Less than 75 % of planned values collected for 1 of the 3 subjects.

Key PK results from the Phase 1b multi-dose study based on NCA methods are shown in Table 7. BTH1677 concentration was generally linear in the range of 1.0 mg/kg to 4.0 mg/kg for multiple iv doses on day 0 and at steady state (days 6–30), based on C_max_, AUC_(0–24)_, AUC_(0-Tau)_, AUC_(0-last)_, and AUC_(0-∞)_ values. PK parameters CL, t_1/2_, V_ss_, and T_max_ were dose independent for BTH1677 on day 0 and at steady state (days 6–30). Among the different treatment groups, comparisons were evaluated on the PK parameters for day 0 versus days 6–30. True K_e_ on day 0 could not be estimated because of protocol design, thus t_1/2_, V_ss_, and CL on day 0 were calculated based on the 0–24-h serum concentration versus time profile. AUC, t_1/2_, and V_ss_ values all tended to be larger on days 6–30 versus day 0.

**Table 7:** Phase 1b multiple-dose non-compartmental pharmacokinetic parameters by treatment group

Pharmacokinetic Parameters^a^										
**Day 0**										
BTH1677 Dose (mg/kg)	Infusion Time (hr)	N	AUC_(0–24)_ (hr*mcg/mL)		AUC_(0-∞)_^b^ (hr*mcg/mL)	CL ^b^ (mL/h)	C_max_ (mcg/mL)	t_1/2β_^b^ (hr)	T_max_^c^ (hr)	V_ss_^b^ (mL)
1	1.0	3	100.64 ± 8.92		132.97 ± 26.84	522.90 ± 121.60	12.65 ± 2.89	13.83 ± 3.97	2.00 ± 1.73	7890.09 ± 575.59
2	1.0	3	181.17 ± 24.35		214.98 ± 31.19	619.83 ± 123.40	36.82 ± 4.73	12.33 ± 1.88	1.08 ± 0.14	6895.74 ± 1228.75
4	2.0	3	369.70 ± 36.14		448.25 ± 60.18	734.56 ± 139.45	63.32 ± 16.48	11.66 ± 4.06	2.50 ± 0.00	8966.61 ± 2812.58
**Day 6–30 (Steady State Days)**										
BTH1677 Dose (mg/kg)	Infusion Time (hr)	N	AUC_(0-λ)_ (hr*mcg/mL)	AUC_(0-last)_ (hr*mcg/mL)	AUC_(0-∞)_ (hr*mcg/mL)	CL_ss_ (mL/h)	C_max_ (mcg/mL)	t_½β_ (hr)	T_max_^c^ (hr)	V_ss_ (mL)
1	1.0	3	134.17 ± 36.10	332.57 ± 145.99	335.20 ± 146.61	528.95 ± 146.83	17.06 ± 2.92	80.97 ± 48.04	1.42 ± 0.52	20,385.20 ± 3273.42
2	1.0	3	230.85 ± 25.20	615.85 ± 120.44	624.57 ± 117.06	572.11 ± 79.72	31.93 ± 3.96	109.51 ± 21.80	1.50 ± 0.50	27,223.33 ± 4208.05
4	2.0	2^d^	639.20 ± 99.43	1483.88 ± 210.62	1494.10 ± 213.00	512.61 ± 158.06	78.07 ± 21.57	95.16 ± 4.93	2.62 ± 0.53	21,986.52 ± 15,281.20

*AUC*_(*0–24*)_ area under the serum concentration versus time curve from time 0 to 24 h after the end of the infusion, *AUC*_(*0-last*)_ area under the serum concentration versus time curve from time 0 until last measurable concentration, *AUC*_(*0-λ*)_ area under the serum concentration versus time curve from time 0 extrapolated to tau, *AUC*_(*0-∞*)_ area under the serum concentration versus time curve from time 0 extrapolated to infinity, *CL* clearance, *CL*_*ss*_ total clearance at steady state, *C*_*max*_ maximum concentration, *t*_*1*/*2*β_ terminal elimination half-life, *T*_*max*_ time of maximum concentration, *V*_*ss*_ steady state of volume distribution

^a^Mean values ± standard deviation.

^b^True K_e_ (defined as rate constant [−slope] from the best-fit, linear regression line of the terminal log-linear serum concentration-time points) could not be estimated for Day 0; parameters were calculated based on the 0–24 h serum versus time profile.

^c^T_max_ was calculated from the starting time of infusion (Time = 0 h) on Day 0.

^d^One subject who did not complete the 7 days of treatment and had less than the required 75 % of data for the day 6–30 PK analysis was excluded.

## Discussion

BTH1677 is a novel beta glucan PAMP being developed for the treatment of cancer in combination with tumor-targeted antibodies, as well as anti-angiogenic and checkpoint inhibitor antibodies. Here, the results of initial safety and tolerability studies of BTH1677 administered alone in healthy volunteer subjects are reported.

In healthy volunteer subjects, the safety and tolerability of single doses and of 7 consecutive daily doses of BTH1677 were investigated by monitoring AEs as well as changes in clinical laboratory parameters, physical exam findings, vital signs, and ECG responses. After adjusting for infusion rate, single doses of BTH1677 up to 6 mg/kg and repeated doses of BTH1677 up to 4 mg/kg (highest doses tested) demonstrated an acceptable safety profile and were well tolerated based on the aforementioned evaluations. Regarding the infusion reactions, it is difficult to distinguish whether the administration of diphenhydramine or simply cessation of infusion (i.e., fluid volume distribution) was responsible for resolution. All observed AEs were mild or moderate in intensity and no DLTs were observed. Although the maximum tolerated dose (MTD) was not reached, an increase in the total number of treatment-related AEs between the 4-mg/kg (2-h infusion) dose group and the 6-mg/kg dose group in the Phase 1a study suggested that higher doses may approach a MTD and, hence, the maximum dose chosen for the Phase 1b multi-dose study was 4 mg/kg.

The PK evaluations in the single-dose study demonstrated linearity with dose and a t_1/2_ of approximately 30 h. The multi-dose PK evaluations demonstrated linearity with dose; however, AUC, t_1/2_ and V_ss_ values tended to be larger on days 6–30 versus day 0. In particular, mean t_1/2_ values were noticeably different across studies following single-dose administration, with mean values ranging from 23.36 h to 32.75 h when blood samples were collected over 168 h in the Phase 1a study, and from 11.66 h to 13.83 h when blood samples were collected over only 24 h in the Phase 1b study. Additionally, mean t_1/2_ values under steady-state conditions (after 7 daily consecutive doses of BTH1677) were noticeably different than those observed under single-dose conditions, with mean t_1/2_ values ranging from 80.97 h to 109.51 h when blood samples were collected over 24 days (Phase 1b study). When using NCA methods, if blood is sampled over an insufficient time span, the apparent terminal elimination phase may be representative of a distribution half-life, as opposed to the true elimination half-life. As such, extending the timing of the blood sampling strategy may be expected to result in different assessments of the terminal elimination phase, as was observed at the first dose in the Phase 1b study (t_last_ = 24 h, t_1/2_ = 11.66–13.83 h), the only dose of the Phase 1a study (t_last_ = 168 h, t_1/2_ = 23.36–32.75 h), and the final dose of the Phase 1b study (t_last_ = 24 days, t_1/2_ = 80.97–109.51 h). Overall, the above results suggest that the true elimination phase of BTH1677 was not well characterized using NCA methods and in future studies additional models will be explored.

There were no clear dose correlations of AE occurrence or AE type with PK parameters in the multi-dose study, but in the single-dose study (excluding the 4 mg/kg 1-h infusion group, which prompted infusion rate modification for the 4 mg/kg and 6 mg/kg doses), the greatest number of AEs occurred in the 6-mg/kg group, which also had the greatest exposure to BTH1677.

Based on the favorable safety and tolerability results observed in the above described safety studies in healthy volunteer subjects, Phase 2 investigations dosing BTH1677 in combination with anti-tumor monoclonal antibodies have proceeded in cancer patients [22–26] and will be reported separately.
